# Development and application of nanomaterials, nanotechnology and nanomedicine for treating hematological malignancies

**DOI:** 10.1186/s13045-023-01460-2

**Published:** 2023-06-23

**Authors:** Jinxin Li, Qiwei Wang, Yingli Han, Lingli Jiang, Siqi Lu, Beini Wang, Wenchang Qian, Meng Zhu, He Huang, Pengxu Qian

**Affiliations:** 1 Center for Stem Cell and Regenerative Medicine and Bone Marrow Transplantation Center of the First Affiliated Hospital, Zhejiang University School of Medicine, Hangzhou, 310058 China; 2 Liangzhu Laboratory, Zhejiang University Medical Center, 1369 West Wenyi Road, Hangzhou, China; 3 Institute of Hematology, Zhejiang University and Zhejiang Engineering Laboratory for Stem Cell and Immunotherapy, Hangzhou, 310058 China; 4 Bone Marrow Transplantation Center, The First Affiliated Hospital, Zhejiang University School of Medicine, Hangzhou, China

**Keywords:** Hematological malignancies, Nanomedicine, Targeted drug delivery, Immunotherapy, Hematopoietic stem cell transplantation

## Abstract

Hematologic malignancies (HMs) pose a serious threat to patients’ health and life, and the five-year overall survival of HMs remains low. The lack of understanding of the pathogenesis and the complex clinical symptoms brings immense challenges to the diagnosis and treatment of HMs. Traditional therapeutic strategies for HMs include radiotherapy, chemotherapy, targeted therapy and hematopoietic stem cell transplantation. Although immunotherapy and cell therapy have made considerable progress in the last decade, nearly half of patients still relapse or suffer from drug resistance. Recently, studies have emerged that nanomaterials, nanotechnology and nanomedicine show great promise in cancer therapy by enhancing drug targeting, reducing toxicity and side effects and boosting the immune response to promote durable immunological memory. In this review, we summarized the strategies of recently developed nanomaterials, nanotechnology and nanomedicines against HMs and then proposed emerging strategies for the future designment of nanomedicines to treat HMs based on urgent clinical needs and technological progress.

## Background

**Fig. 1:**
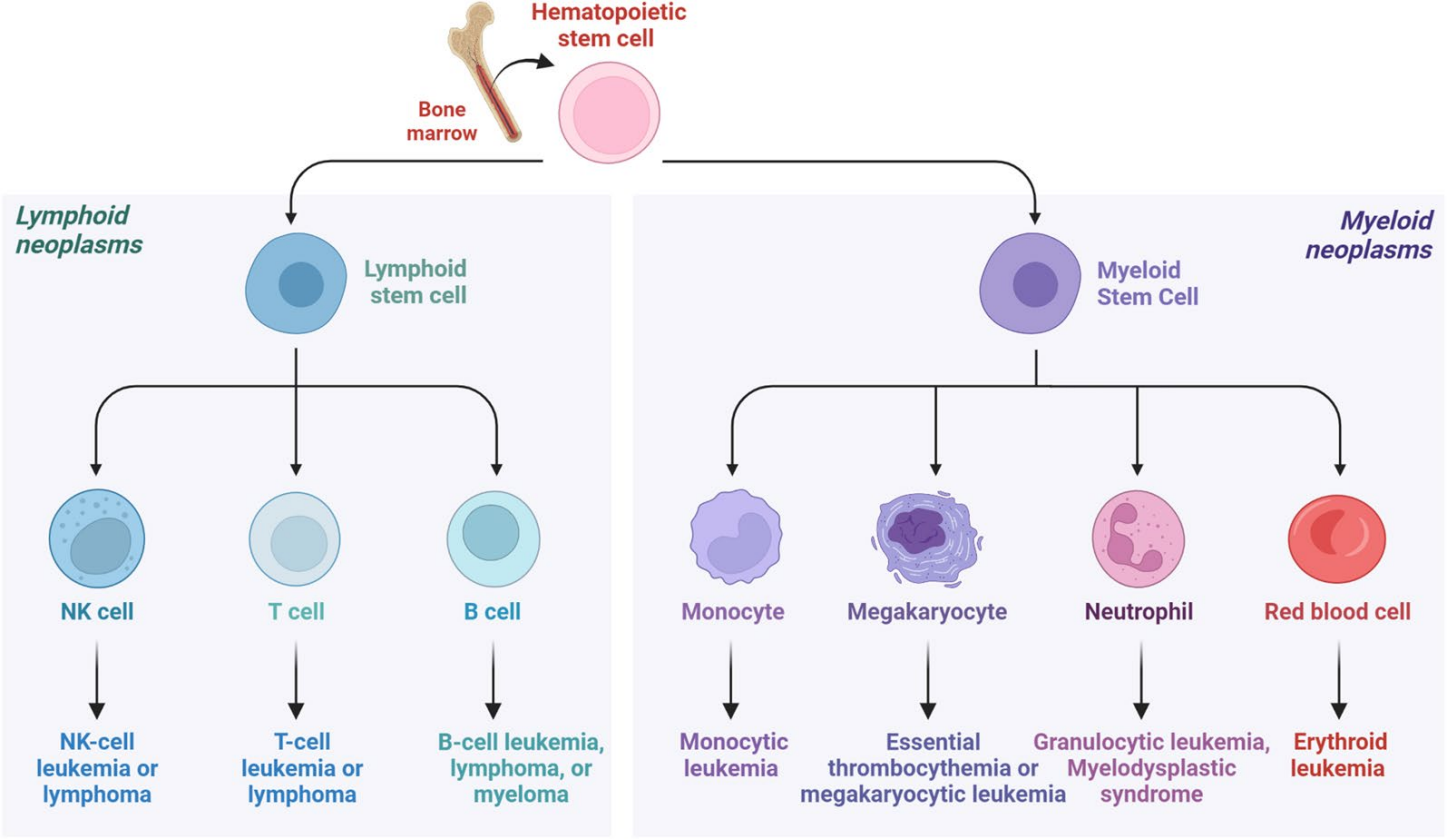
The main subtypes of hematological malignancies

Hematopoietic malignancies (HMs) are originated from hematopoietic system, mainly including leukemia, lymphoma, multiple myeloma (MM) and myelodysplastic syndromes (MDS) (Fig. 1), which increase mortality and morbidity and seriously threaten human health [1, 2]. However, due to the lack of understanding of the pathogenesis and the lack of effective drugs, the 5-year overall survival rate of HMs is extremely low, causing serious economic and life burdens to patients [3, 4]. In terms of pathogenesis, leukemia and MDS are mainly caused by the malignant clonal proliferation of hematopoietic stem/progenitor cells (HSPCs) in the bone marrow [5–10]. MM also originates from bone marrow, but from malignant proliferation of plasma cells [11]. The pathogenesis of lymphoma has not been fully elucidated and virus and abnormal cell metabolism are important trigger factors [12].

Current clinical strategies for treating HMs include radiotherapy, chemotherapy, targeted therapy and hematopoietic stem cell transplantation (HSCT) [13, 14]. Chemotherapy is the most basic therapy for treating HMs, and the classic first-line therapy of “3 + 7” regiment (daunorubicin and cytarabine) against leukemia has been in clinical practice for decades since 1973 [15]. The combination of chemotherapy and targeted drugs plays an increasingly important role in the treatment of HMs. The combination regimen of rituximab, cyclophosphamide, doxorubicin, hydrochloride, vincristine sulfate and prednisone (R-CHOP) has become the current standard treatment regimen for treating B cell lymphoma [16]. Recently, the DAV regimen (doxorubicin, cytarabine combined with venetoclax) has shown good promise in clinical trials [17]. HSCT is the most promising therapy to completely cure HMs, but the scarcity of donor cells always restricts the effectiveness of transplantation [18]. As for immunotherapy, researchers obtained positive results utilizing a combination of nivolumab (PD-1 inhibitors) and AZA treatment to treat patients with refractory AML whose expression levels of PD-1 and PD-L1 increased constantly [19]. Other clinical studies have demonstrated that nivolumab combined with ibrutinib (BTK inhibitors) can effectively treat refractory CLL [20]. Most clinical trials are still in the initial stage. Chimeric antigen receptor-modified T cell (CAR-T) therapy has achieved remarkable progress in treating B cell-derived malignancies, the severe side effects and high expense limit its clinical application [21]. Therefore, HMs require more effective and de novo therapeutic approaches.

**Fig. 2:**
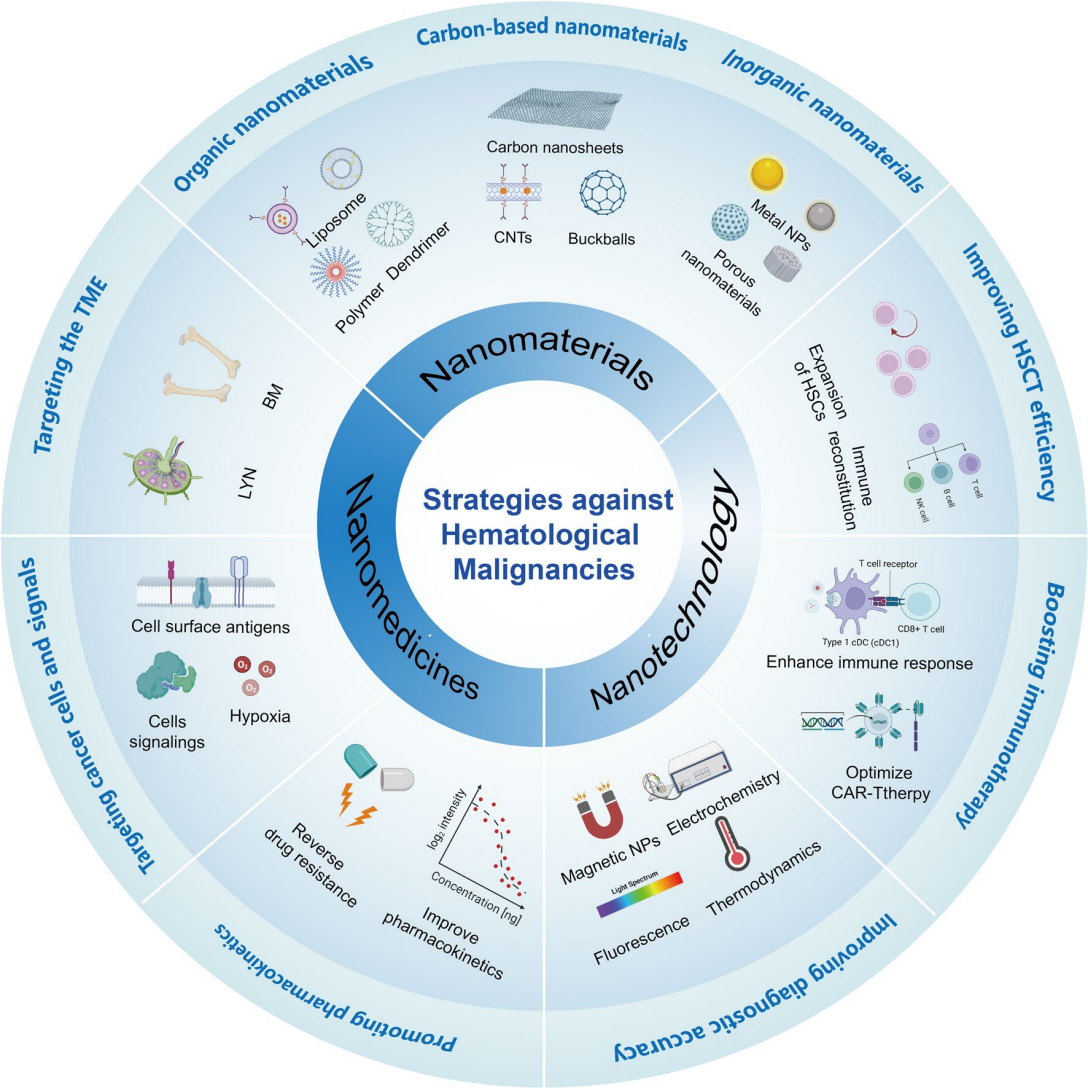
The main content of this review and proposed strategies

Nanomedicine based on nanomaterials and nanotechnology show great advantages in cancer treatment and diagnosis [22, 23]. Nanotechnology can be used to rapidly identify cancer cells from complex or rare samples, which improve the precision and accuracy of diagnosis [24]. In cancer treatment, unlike traditional drugs, nanodrug delivery systems can enable targeted delivery of drugs, increase drug accumulation at the tumor site, achieve controlled drug release and reduce systemic toxicity [25]. Moreover, nanomedicine shows advantages in overcoming drug resistance and enhancing the immune response [26]. Recurrence is the most intractable problem in hematological malignancies. Therefore, the usage of nanomedicine to enhance the body’s immune response and immunological memory is an effective strategy for the long-term control of HMs. Clinically, the application of HSCT after chemotherapy is a common strategy for treating HMs. However, the lack of donor HSC cells is an important factor limiting the success rate of HSCT [27]. Nanoscaffold structures or modified hydrogels show great promise in the expansion of stem cells [28–30]. Ex vivo expansion systems based on nanomaterials or hydrogels can be used to increase the number of HSCs without impairing the hematopoietic capacity, thus improving the effectiveness of HSCT [18]. In this review, we first introduce the main subtypes and pathogenesis of HMs and current therapies used to treat these diseases. Then, we systematically propose the strategies for treating HMs (Table 1; Fig. 2) and provide prospects for the future design of HMs nanomedicines.

**Table 1:** Strategies for designing and constructing nanomedicine against HMs

Clinical needs	Strategies	Nanomaterials or carriers
Optimize the HSCT	I. Expansion of HSCs ex vivo	Nanoparticles, nanofibers, hydrogels
	II. Promote immune reconstitution	Bionic hydrogels
Boost immunotherapy	I. Improve antigen presentation	PLA NPs, hydrogels
	II. Improved CAR-T therapy	PGA polymer
Targeted drug delivery	I. Targeting TME	Bionic vesicles, living or dead cells
	II. Targeting cancer cells	Organic or inorganic nanoparticles
	III. Targeting intracellular signals	Organic or inorganic nanoparticles
	IV. Reverse multidrug resistance	Organic or inorganic nanoparticles

*HSCs* hematopoietic stem cells, *TME* tumor microenvironment, *PLA NPs* poly (lactic acid) nanoparticles, *PGA* poly (glutamic acid)

## Nanomaterials applied in construction of nanomedicine against HMs

### Nanomedicine and nanomaterials in anticancer therapy

Nanomedicine refers to use of nanotechnology to make drugs into nanoparticles with particle size between 1 and 100 nm, or the combination of appropriate carrier materials with bulk drugs to form nanoparticles with nanometer scale and the final pharmaceutical preparation [31]. Compared with traditional free small molecule drugs, nanomedicine has longer blood circulation time, stronger targeting and more effective therapeutic effect [22]. Nanomaterials, with a particle size about 1–100 nm, have special properties different from macroscopic materials and are widely used in medicine and pharmaceutical fields. Nanomaterials are mainly divided into organic and inorganic nanomaterials [25]. Currently, FDA-approved or clinically studied nanomedicine against HMs is mainly based on organic nanomaterials, such as liposomes and polymer micelles (Table 2). Inorganic nanomaterials are usually studied more widely in the diagnosis of cancer [32]. In this chapter, we will introduce these various nanomaterials used in HMs in detail.

**Table 2:** Representative FDA-approved drugs or clinical trials of nanomedicine against HMs

Product name	Carrier	Drug	Status	Outcome or State	Identifier
*Leukemia*					
Marqibo kit	Liposome	Vincristine	Approved		
Vyxeos	Liposome	Ara-C, DOX	Approved		
Oncaspar	PEG	Pegaspargase	Approved		
MB-106	Liposome	Annamycin	Phase 1	80% Overall Response Rate in Final Cohort of Phase 1	NCT05319587
*Lymphoma*					
2022–0453	Liposome	MTX	Phase 2	P2, Second Affiliated Hospital, School of Medicine, Zhejiang University *N* = 45, Recruiting	NCT05495100
*MM*					
MCC-17814	Liposome	DOX	Completed	P2, H. Lee Moffitt Cancer Center and Research Institute	NCT02186834
	Liposome	MTX	Phase 1	P1, CSPC Zhongnuo Pharmaceutical (Shijiazhuang) Co., Ltd. *N* = 60, Recruiting	NCT05052970
*MDS*					
TLK199 HCl	Liposome	Ezatiostat, HCL	Phase 2	P2, Telik, *N* = 65, Completed	NCT00035867
NCI-2018–01812	Liposome	Ara-C, DOX	Phase 2	P2, M.D. Anderson Cancer Center *N* = 50, Recruiting	NCT03672539
AEB1102	PEG	Human arginase I	Phase 2	P2, Aeglea Biotherapeutics, *N* = 29, Completed	NCT02732184

From www.clinicaltrials.gov

*MTX* mitoxantrone, *HCL* hydrochloride

### Liposome

Liposome is the most widely used drug delivery system in biomedical research and clinical application [33–35].

Liposomes are widely used and studied in the treatment of blood cancer [36, 37]. CPX-351 and Marqibo (R) are the well-known liposome drug for the treatment of leukemia [38–40]. Cytarabine and doxorubicin are the main components of CPX-351 [41]; compared with traditional free drugs, CPX-351 has better pharmacokinetic characteristics and brings more significant clinical benefits to patients [40, 42]. Liposomes loaded only with cytarabine also showed good results in the treatment of leukemia [43]. Doxorubicin is another important chemotherapy drug for leukemia, and its liposome drugs have been widely studied and reported [44]. Doxorubicin liposomes can significantly improve the clinical therapeutic effect of leukemia patients [45, 46]. In addition to traditional chemotherapy drugs, liposomes are also used to coat other drugs, such as glucocorticoids [47, 48], AP9-cd [49], ceramides [50, 51], vincristine [52], annamycin [53] and other novel drugs. Nucleic acid drugs show great promise in the treatment of leukemia [54, 55], and liposomes are also used to deliver nucleic acid drugs to treat CML leukemia [56].

Surface-modified liposomes are also widely studied. Antibody-modified liposomes can be used to specifically target leukemia cells [57]. Myers et al*.* reported a liposome modified with CD19 antibodies for the treatment of B cell leukemia [58]. Shao et al*.* reported a hyaluronic acid-modified liposome for the treatment of leukemia [59]. Combined with the latest nanotechnology, the controlled release ability of liposomes is further improved. Gui and colleagues developed a temperature and light-controlled liposomes drug for controlled release of chemotherapeutic drugs [60].

Shi et al*.* constructed a liposome with dual targeting capability to remove leukemia stem cells and minimal drug residues [61].

### Polymer

Polymer-based nanoparticles are widely used in preparation of nanomedicine [62]. Polymer nanomaterials used in HMs mainly include PLA, PLGA and chitosan [63].

Poly(lactic-co-glycolic acid) (PLGA)-based nanomaterials are widely used in drug carriers. Noureldien et al*.* reported a PLGA-based nanodrug for the targeted treatment of AML [64]. Khan et al*.* constructed a PLGA nanodrug loaded with bendamustine, which can significantly enhance the toxicity to leukemia cells [65]. In addition, polymer nanoparticles can also be used to reverse drug resistance [66]. Leung et al*.* also constructed curcumin-loaded PLGA-based nanoparticles (Cur-PLGA NP) for the treatment of Jurkat leukemia cells [67]. Su et al*.* constructed a erythrocyte membrane cloaked, ATO-loaded PLGA nanoparticle for APL treatment and to reduce the side effects of arsenic agents [68]. Venkatpurwar et al*.* demonstrated the biosafety of PLGA as a delivery carrier [69]. Wang et al*.* demonstrated that PLGA nanoparticles encapsulated with Iguratimod could induce MM cell death, which could be used as a potential therapy for MM [70].

Chitosan nanoparticles are widely applied in drug delivery carriers. Gong et al*.* reported a chitosan-based, self-assembled nanomedicine for the controlled release of 6-MP and treatment of AML [71]. Derakhshandeh demonstrated that Gemcitabine in the form of chitosan nanoparticles can increase intestinal transport by three–fivefold [72]. Similarly, Alassaif’s work showed that chitosan-coated anthraquinone nanoparticles can significantly enhance the toxicity against leukemia cell HL-60 [73]. Sarangapani’s work demonstrated that chitosan nanoparticles can selectively kill leukemia cells by clearing glutathione and elevating ROS [74]. Similarly, Saravanakumar et al*.* showed that the preparation of zinc–chitosan nanoparticles (Zn-CSNPs) by linking zinc with chitosan could also enhance the toxicity against leukemia cells [75]. Termsarasab et al*.* constructed a PEGylated, chitosan-based nanodelivery system for prolonging the blood circulation time of DOX [76].

### Other biomacromolecules as delivery carriers

Gigli’s study showed that simultaneous delivery of two polymer nanoparticles containing different drugs consistently down-regulates CML cancer development [77]. Boto et al*.* have constructed a light-induced polymer nanoparticle to enhance leukemia treatment [78]. To overcome the delivery barrier of therapeutic enzymes, Blackman et al*.* have built a polymer-based self-assembly-based nanodrug to deliver leukemia treatment drugs [79]. Ma and colleagues used protein-based scaffold as nanocarriers to deliver antitumor peptides for CML therapy [80]. Li et al*.* reported a carrier-free catanionic drug-derivative nanodrug for the treatment of leukemia [81].

### Carbon-based nanomaterials

#### Carbon-based nanomaterials as delivery carrier

Carbon-based nanomaterials are widely studied as drug delivery carriers in anticancer research [82–84]. Carbon-based nanomaterials such as fullerenes, carbon nanotubes, graphene and carbon nanodiamond all have widely application prospects in the treatment of tumors or drug delivery [85, 86].

One-dimensional carbon nanotubes are widely used as drug delivery vehicles. Ruibin et al*.* reported a P-gp antibody (anti-P-gp) functionalized water-soluble single-walled carbon nanotubes (Ap-SWNTs) loaded with doxorubicin (Dox) to kill MDR human leukemia cells K562 [87]. Carlos H. Villa et al*.* reported the use of single-walled carbon nanotubes (SWNT) as antigen carriers to improve the immune response to weak immunogenic peptides [88]. In vitro, peptide-SWNT constructs were rapidly internalized into APCs cells (dendritic cells and macrophages) in a dose-dependent manner.

Two-dimensional carbon nanomaterials such as graphene nanosheets also have been applied in targeted delivery of anticancer drugs [89]. Roy et al*.* reported a RGO/Ag composite nanoparticle that has a strong anticancer activity against KG-1A cell line [90]. The increase in ROS induced apoptosis in KG-1A cells exposed to nanocomposites may be the cause of cell death. Three-dimensional carbon nanomaterials, nanodiamonds (NDs), also be used as drug delivery platform for cancer treatment. Man et al*.* synthesized ND vectors capable of loading chemotherapeutics and gene delivery and applied them to the treatment of drug-resistant leukemia [91].

#### Carbon nanomaterials with intrinsic anticancer properties

Inorganic nanoparticles are also widely used in the treatment of leukemia [92]. In consideration of carbon nanomaterials that can regulate cell adhesion and guide cell fate, Wang et al*.* screened a variety of one- and two-dimensional carbon materials and unexpectedly found that GDYO showed a strong killing effect on DNMT3A-mutant AML cells [93]. By analyzing the GDYO-binding proteome, they found that GDYO specifically binds to two membrane proteins, ITGB2 and MRC2, which are highly expressed in DNMT3A-mutant AML cells, thereby increasing the intake of GDYO in DNMT3A-mutant AML cells. After entering the cell, GDYO interferes with the normal assembly of F-actin cytoskeleton through direct interaction with actin and ultimately leads to cell death. Finally, they verified the in vitro efficacy of GDYO in DNMT3A mutated AML and the biosafety of GDYO.

GDYO also inhibits the growth of lymphoma. Lymphoma is a type of solid tumor originating in the lymphatic system. Li et al*.* reported that GDYO nanosheets can simultaneously kill lymphoma cancer stem cells and remodel tumor microenvironment, thus inhibiting the growth of lymphoma [94]. Mechanistically, GDYO treatment significantly reduced the number of cancer stem cells and the level of Mif-Ackr3 signaling from tumor cells to cancer-associated fibroblasts (CAFs), resulting in a decrease in inflammatory cytokines secreted by CAFs in the microenvironment, which further led to a decrease in the number of Tregs, thereby remodeling the immunosuppressive and inflammatory microenvironment.

### Silicon-based nanomaterials

Porous silica nanomaterials have great prospects in biomedicine as drug delivery carriers due to porous and good drug adsorption properties [95–98]. Durfee et al*.* used MSN nanoparticles as the carrier to construct nanodrugs for active targeting of leukemia cells to deliver drugs [99]. Tao et al*.* prove that mesoporous silica microparticles can be used to enhance the toxicity of anticancer platinum drugs [100].

### Arsenic-based nanoparticles

Arsenic, as a traditional Chinese medicine, mainly includes arsenic and realgar. Arsenic trioxide, the active ingredient in arsenic, was first purified in the 1970s and used to treat APL, increasing the cure rate to more than 90%. However, the high toxicity and side effects limit the further anticancer application of arsenic agents. In view of this, Peng et al*.* prepared ATO into FA-HSA-ATO nanodrug to reduce the toxic and side effects of arsenic agents and improve the efficacy of targeted therapy [101]. Similarly, Richard et al*.* reported that nanoencapsulating ATO can significantly improve the therapeutic efficacy of leukemia and reduce the toxic side effects on ovaries [102]. However, the clinical pharmacokinetics and toxicity of arsenic agents also need to be carefully studied to prevent excessive side effects [103, 104].

Realgar is another important arsenic agent, mainly composed of As_4_S_4_. Research evidence confirms that realgar has a similar therapeutic effect on APL as ATO, but it is insoluble in water and most organic reagents, resulting in poor bioavailability and limiting its clinical application. Inspired by nanodrugs, Wu et al*.* demonstrated that preparation of realgar into nanoparticles can increase bioavailability and enhance toxicity to cancer cells [105]. Shi et al*.* further confirmed the important role of caveolin-1 (Cav-1), a principal constituent protein of caveolae, in mediating the absorption of realgar nanoparticles by leukemia cells [106]. In addition, it was shown that co-delivering realgar with other drugs also enhanced the antileukemia effect [107].

### Metal nanoparticles

#### Gold nanoparticles

Gold nanoparticles are widely used as drug delivery carriers due to their good stability and adsorption ability. Molotkova and colleagues reported using gold nanoparticles as carriers to deliver Bosutinib, a TKI, for CML therapy [108]. Simon et al*.* constructed nanomaterials based on gold nanoparticles to deliver small molecule inhibitors of FLT3 for the treatment of AML [109]. On this basis, Suarasan et al*.* constructed gelatin-coated gold nanoparticles as carriers of FLT3 inhibitors for the treatment of acute myeloid leukemia [110].

#### Silver nanoparticles

Silver nanoparticles have unique optical, electrical and catalytic properties and can be used in optical materials, battery electrodes and catalysts. In addition, nanosilver also shows certain application value in antifungal, anti-viral, anti-inflammatory and anti-thrombosis and antitumor and promoting wound healing [111]. There are a large number of reports on the application of nanosilver in the treatment of leukemia. In this section, we will summarize it and select representative studies for elaboration.

Foldbjerg et al*.* found that PVP-coated nanosilver can induce apoptosis and necrosis of THP-1 cells in a dose- and time-dependent manner [112]. This effect may be mediated by the increase in ROS caused by nanosilver. Similarly, Rajendrana et al*.* found that Ag NPs prepared from FA and CHA exhibited significant anticancer activity on K562 cells at a lower concentration due to ROS production and DNA fragmentation [113]. Hemmati et al*.* reported a simple, cost-effective and green method to synthesize Ag NPs nanomedicine using chitosan as a reducing agent and stabilizer [114]. These silver nanoparticles ranged from 20 to 30 nm, showing toxicity to 32D-FLT3-ITD and murine leukemia C1498 cell lines. Ag NPs chitosan composite may be used as a chemotherapeutic drug for the treatment of myeloid leukemia.

Interestingly, the shape of the nanoparticles also affects their toxicity to tumor cells. Sakaguchi et al*.* showed that the shape of Ag nanomaterials plays an important role in its anti-proliferative activity, and the activity of anti-proliferative silver nanomaterials highly depends on its nanostructure [115]. The Ag nanoplates have significantly higher anti-proliferative activity against human promyelocytic leukemia HL-60 cells than spherical nanoparticles. The triangular Ag nanoplates can induce apoptosis but are located in the same subcellular compartment as the spherical Ag nanoparticles. The research helps to design and optimize silver nanostructures for cancer treatment.

#### Magnetic nanomaterials

Magnetic nanomaterials are an important category of nanomaterials, typically ranging in size from 1 to 100 nm. As a new type of functional material, magnetic nanoparticles have a broad application prospect in the field of magnetic materials and bioengineering [116]. Due to its special properties, it is easy to separate under the action of external magnetic field, which brings great convenience to the separation of target biological products. And because it can be rapidly enriched in magnetic field, it provides the possibility for targeted drug delivery. At present, magnetic nanoparticles, as a carrier of targeted drug delivery and a tool of bioseparation technology, have received extensive attention and research.

Gang et al*.* reported a Fe_3_O_4_-PLA nanocomposite material for drug delivery and explored the potential application of the daunorubicin (DNR) to the drug-resistant leukemia K562 cells [117]. The new nanocomposite can promote the interaction between the anticancer drug DNR and the targeted cancer cells and strengthen the accumulation of anticancer drugs in a single leukemia cell. Similarly, Chen et al*.* linked magnetic Fe_3_O_4_ nanoparticles with Homoharringtonine (HHT), a natural cephalotaxine alkaloid, for tumor therapy, further demonstrating the potential of magnetic Fe_3_O_4_ nanoparticles as a carrier [118].

Anisotropic nanoparticles have a longer blood circulation lifespan than previous isotropic nanoparticles. Based on this strategy, Xiong et al*.* fabricated superparamagnetic anisotropic nanocomponents (SAN) and loaded vincristine (VCR) to form VCR-SAN nanoparticles [119]. VCR-SANs have rapid and sustained release behavior, longer blood circulation and tissue distribution in the body and have strong antileukemia ability. Through in vivo and in vitro experiments, compared with the same dose of isotropic nanocomponent drugs, superparamagnetic anisotropic nanocomponents loaded with VCR can treat leukemia more effectively [119]. Díez et al*.* reported the simple and effective combination of IONP nanoparticles with bile acid–cisplatin derivatives to use as antitumor drugs and demonstrated its cytotoxicity to T cell leukemia (Jurkat) cells [120].

Magnetic nanoparticles also show good properties in drug loading and delivery. El-Boubbou et al*.* have developed an iron oxide nanoformulation loaded with the anticancer drug Doxironide, which can be used as a selective drug carrier for different types of AML [121]. Musawi et al*.* prepared a chitosan-coated magnetic nanoparticle (CS-SPION) and loaded the anticancer drug paclitaxel, FA-CS-PTX-SPION, through reverse microemulsion technology [122]. FA-CS-PTX-SPION is spherical, with an average diameter of 90 ± 15 nm. Cytotoxicity experiments on cancer cells (K562) and normal cells (GK-5) showed that FA-CS-PTX-SPION can significantly induce apoptosis on cancer cells, while there is no obvious toxic effect on normal cells.

In other blood cancer, Xia et al*.* constructed nanoparticles using magnetic Fe_3_O_4_ nanoparticles as the carrier to deliver 2-methoxyestradiol for MDS treatment [123].

#### Other metallic nanoparticles

In addition to the commonly used metallic nanoparticles as delivery carriers, other metal nanoparticles such as titanium oxide, calcium and palladium have also been used in leukemia treatment research.

Nanotitanium dioxide (TiO_2_) is a nanomaterial widely used in medicine and life sciences. Song et al*.* reported the use of highly reactive TiO_2_ nanoparticles combined with daunorubicin to inhibit the MDR resistance of leukemia K562 cells [124]. The principle of this strategy is to increase the intracellular concentration of targeted drugs through the synergistic effect of TiO_2_. Regarding the toxicity of TiO_2_ nanoparticles to blood cells, Cui et al*.* found that TiO_2_ nanoparticles are not toxic to macrophages THP-1 within a concentration range as high as 220 µg/mL and showed that this is for any safe nanotechnology product [125]. It should become a necessary requirement.

Palladium nanoparticles have gained attention in precious metal nanoparticles due to their wide application in materials science and medicine. Li et al*.* reported the biological production of palladium nanoparticles (Pd NPs) using the aqueous leaf extract of *Geranium Geranium to* kill leukemia cells [126]. The in vitro cytotoxicity study of Pd NPs encapsulated by *Geranium Geranium* extract on human leukemia cell line (K562) showed a dose-dependent cytotoxicity. The green synthetic Pd NPs will bring new opportunities to the biomedical field. Kaur et al*.* developed a dual-functional nanocarrier using palladium [127]. The nanocarrier has anticancer and antibacterial activities and is prepared by a ligand insertion method by using cetyltrimethylammonium chloride and palladium chloride in a simple and cost-effective method. The Palladium surfactant shows cytotoxicity to human leukemia HL-60 cells, and the lower IC_50_ value indicated that it has the potential to be used as an anticancer agent.

As for other rare metal-based nanomedicines, Jurcic et al*.* reported a Targeted Alpha-particle Nano-generator Actinium-225 (225Ac)-lintuzumab (anti-CD33) nanomedicine for the treatment of acute myeloid leukemia (AML) [128]. Cerium oxide nanoparticles have been proven to scavenge free radicals and have the potential to be used as disease treatment agents. Patel et al*.* used human monocytic leukemia cells (THP-1) as a model to evaluate the uptake and free radical scavenging activity of nanocerium oxide [129]. The data showed that the internalization of nanocerium oxide in THP-1 cells was significantly increased in a concentration-dependent (10–100 µg/mL) manner. Although no cytotoxicity was observed at these concentrations, nanocerium oxide significantly reduced the amount of reactive oxygen species. This study shows that cerium oxide has therapeutic potential in diseases such as cancer [129].

For other blood cancer, briefly, Chen et al*.* constructed a nanoparticle using cadmium telluride quantum dots as nanocarriers to deliver DOX for MM treatment [130]. Li et al*.* proved that zinc oxide nanoparticles could induce apoptosis of MM cells, which was a potential clinical agent for MM [131].

## Nanotechnology for improving HSCT

### Nanotechnology to promote bone marrow transplantation

#### Expansion of HSCs ex vivo

Thomas first utilized HSCT in leukemia treatment in the 1960s [132]. HSCT is the most promising therapy to cure blood diseases and is usually applied during the remission period of leukemia. After high-dose treatment with cytotoxic drugs or radiation to destroy the abnormal hematopoietic system and cancer cells temporarily and entirely, autogenic or allogenic HSCs are perfused into patients to restore the functional bone marrow and reestablish the hematopoietic and immune systems. Clinically, HSCT is usually performed after remission from chemotherapy in HMs patients. Patients who undergo HSCT also face the risk of recurrence, and severe graft-versus-host disease (GVHD) is also an important factor limiting the effectiveness of HSCT [133].

**Fig. 3:**
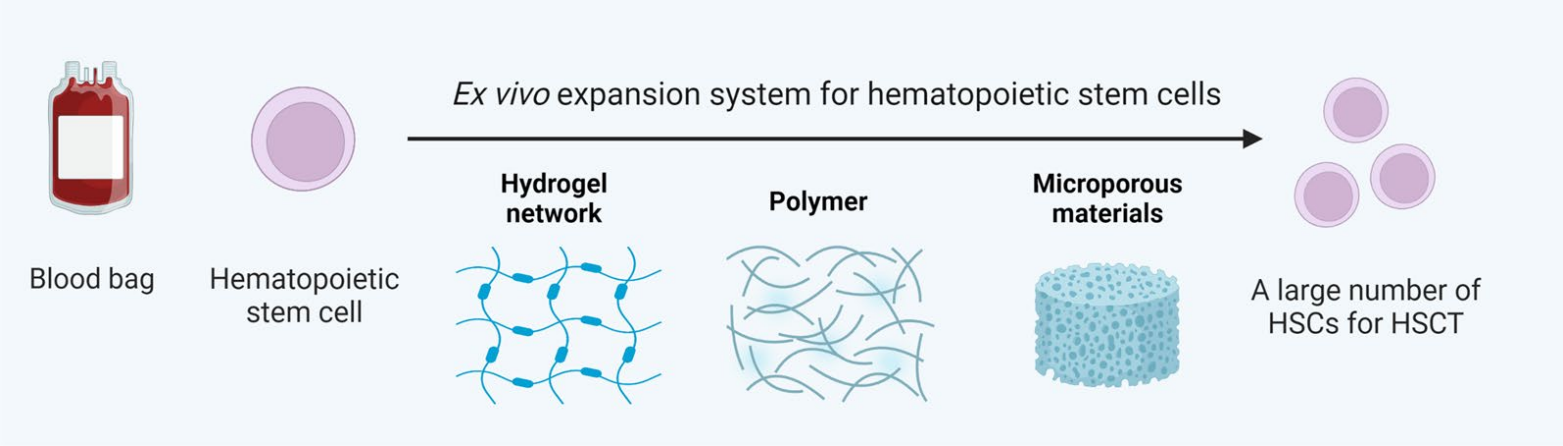
Strategies to expand hematopoietic stem cells ex vivo*.* Using hydrogels, polymer fibers and bone marrow biomimetic materials such as porous materials to obtain a large number of HSCs and improve the efficiency of HSCT

However, the lack of donor HSCs is an important factor limiting the effectiveness of HSCT, so increasing the number of HSCs is an effective strategy for improving HSCT. Ex vivo expansion of stem cells requires more complex conditions than that of mature cells. The use of nanomaterials to expand stem cells has been extensively investigated [134–138]. Specially, nanomaterials hold great promise for maintaining the ex vivo expansion of HSCs (Fig. 3).

The bone marrow microenvironment plays an important role in maintaining the homeostasis and expansion of HSCs [139–141]. Mimicking the bone marrow microenvironment ex vivo is an effective strategy to maintain HSCs [142, 143].

Hydrogel is commonly used for expanding stem cells ex vivo [144]. Gvaramia and his colleagues studied the biochemical and biophysical signals that influence the maintenance and proliferation of hematopoietic stem cells in the form of hydrogels [145]. Bai et al*.* reported an amphiphilic hydrogel that can amplify HSCs [18]. Mechanically, the microenvironment provided by the hydrogel can reduce the ROS level of HSC cells, thus facilitating HSC amplification. In addition, similar work has demonstrated the feasibility of expanding cells with hydrogels [146, 147]. In addition, microcavity arrays also can be used to mimic the bone marrow microenvironment for HSCs expansion [148]. Ferreira et al*.* constructed a fibrin scaffold for the expansion of cord blood hematopoietic stem cells in vitro [149]. Predictably, if the microenvironment is not conducive to the maintenance of the HSC, the HSC will loss of quiescence and self-renewal capacity [150]. Finally, it is also very important to understand the mechanism of the signaling pathway between hematopoietic cells and material matrix [151–154].

Nanoparticles can also promote HSCs amplification in vitro. PVA nanomaterials are widely used in LCD electronic screens, semiconductors and other applications. Wilkinson et al*.* reported that polyvinyl alcohol (PVA) can be used to amplify the number of HSCs [155]. In mouse experiments, the results of transplantation proved that the hematopoietic capacity of expanded HSCs was not affected by PVA treatment. Bari’s work demonstrated that the use of functionalized carbon nanotubes can promote in vitro expansion of human cord blood hematopoietic stem progenitor cells [156]. The mechanism is due to the reduction of mitochondrial superoxides and caspase activity in CD45 + cells. In addition, a variety of nanofibers can also be used to amplify HSCs [157, 158].

#### Promote immune reconstitution after HSCT

After HSCT, the immune system cannot be rebuilt immediately, and thus, patients are susceptible to bacterial infection or external stimulation, finally leading to failure of transplantation. The efficiency of the reconstitution of the immune system after HSCT is an important factor affecting the prognosis of patients. Shah et al*.* reported a method to facilitate T cell reconstruction and immune response after HSCT [159]. This study used an alginate hydrogel to mimic the bone marrow microenvironment, and this artificial scaffold can considerably promote the differentiation and reconstruction of immune cells.

### Enhancing immunotherapy

Immunotherapy is a major milestone in cancer treatment and is the most promising way to completely control cancer [160]. Tumor immunotherapy has a century-old history, including vaccines, cytokines, antibody drugs, immune checkpoint inhibitors, adoptive cell therapy and many other technologies [161]. The earliest immunotherapy can be traced back to Dr. William Colley’s “Colitoxin” in the late nineteenth century. Now, we know that the main component of “Colitoxin” is tumor necrosis factor α (TNF-α). With the advancement in immunology, the discovery of different immune checkpoints has revealed new approaches for antitumor immunotherapy. Blocking immune checkpoints have been found to eventually enhance the antitumor effects of the immune system. Immunotherapies have achieved good progress in cancer treatment [162]. The combination of immune checkpoint inhibitors and chemotherapy or targeted drugs also has achieved good results [163].

Utilizing nanotechnology to enhance immunotherapy holds great promise in cancer research [26]. The application of nanotechnology to enhance the therapeutic effect of immunotherapy for hematologic malignancies shows great prospect for HMs patients. By summarizing the approved or under test nanodrugs for HMs (Table 2), we found that they are mainly focus on enhancing drug delivery, while there are no reports of nanodrugs for activating the immune system. In this section, we will introduce nanodrugs for immunotherapy against hematologic malignancies.

#### Stimulating immune response

The high recurrence rate is a key factor leading to the low five-year survival of leukemia [164]. An effective strategy to prevent the recurrence of leukemia is to promote the body forming a lasting immune memory. Vaccines play an important role in the formation of immunological memory, but traditional bulk vaccines have low immunogenicity and cannot effectively stimulate the body to produce a durable immune response. The encapsulation of traditional antigens into nanoparticles or nanomaterials can considerably boost the immune system’s recognition of antigens [165].

**Fig. 4:**
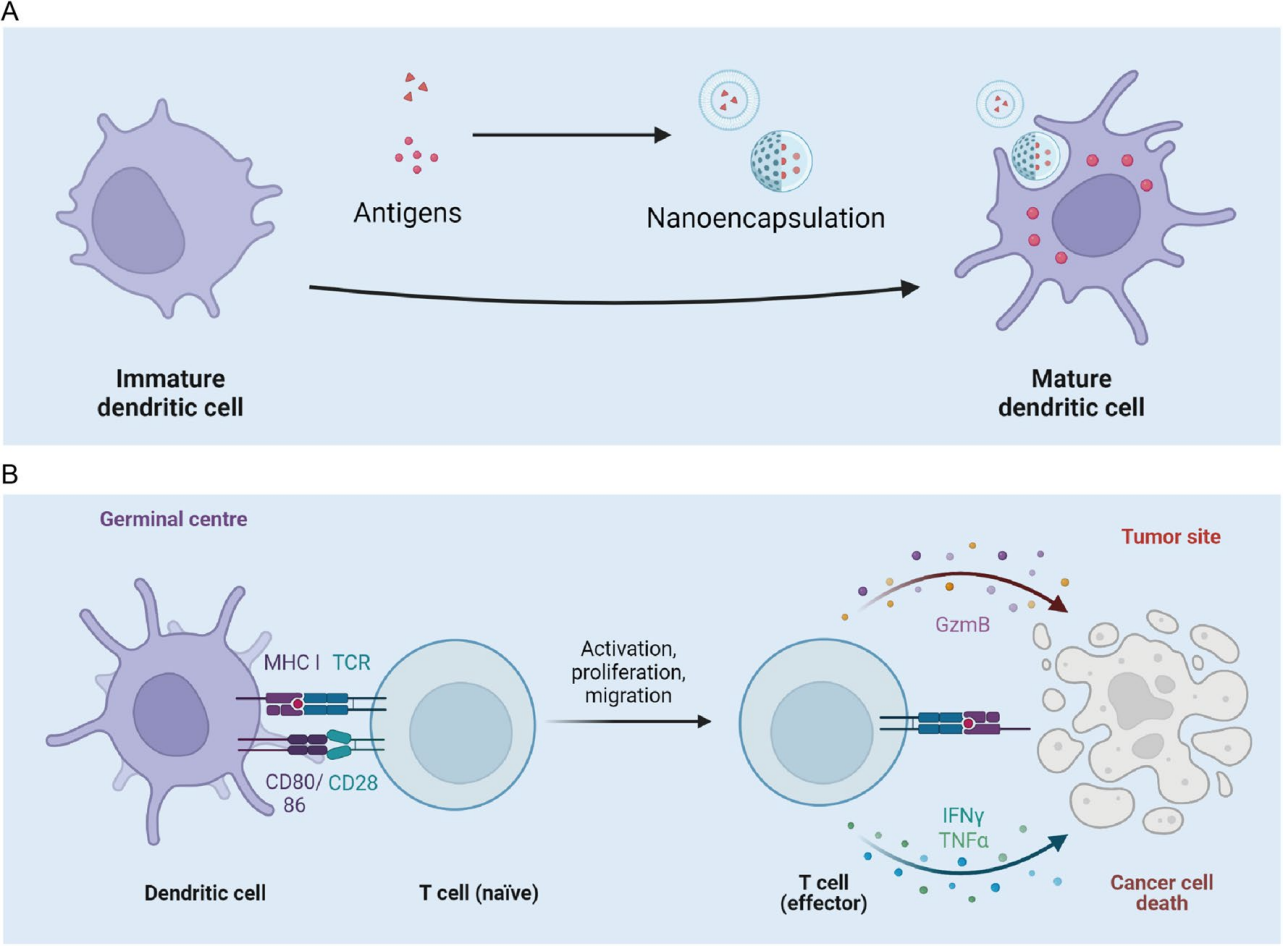
Strategies to boost immunotherapy. **A** Encapsulation or preparation of antigens into the form of nanoparticles can enhance activation of APC cells. **B** Activated APC can enhance the ability of T cells to kill tumor cells

Using biological materials is an effective way to improve the immune system’s recognition of antigens (Fig. 4). Mooney et al*.* reported that a hydrogel-based AML vaccine can produce a long-lasting immune response in the body [166]. The vaccine uses MA-alginate hydrogel as the framework and loaded with leukemia antigen polypeptide WT1126-134 and immune adjuvants inside. In animal experiments, the vaccine can prevent the recurrence of leukemia.

Nanoparticle formulations also improve the immune system’s recognition of antigens. Ma et al*.* reported a therapeutic leukemia vaccine [167]. The vaccine comprises porous PLA microspheres loaded with PD-1 antibody and leukemia antigen polypeptide pE. The vaccine can enhance the APC response at the injection site and recruit more APC cells, enhance the proliferation of T cells in lymph nodes and increase the toxicity of cytotoxic T cells to leukemia cells. Animal experiments show that the vaccine can prolong survival and prevent the recurrence of leukemia in both CDX and PDX leukemia model mice.

Boosting the immune system is also an effective strategy for lymphoma and myeloma. Enhancing the body’s immune response and memory is an effective strategy to prevent recurrence. Islam et al*.* reported a nanomedicine that can enhance the immune response to lymphoma [168]. Regarding myeloma, Bae et al*.* reported a BCMA peptide-engineered nanoparticle for the enhanced clinical treatment of multiple myeloma by enhancing the function of CD8 + cytotoxic T lymphocytes [169]. Please refer to Table 3 for a detailed summary.

**Table 3:** Representative nanomaterials in treating HMs that stimulate the immune system

Nanomaterials	Payload	Ind.	Outcome	References
PLA microspheres	PD-1 antibody and leukemia antigen polypeptide pE	Leu	Enhance the APC response, recruit more APC cells, enhance the proliferation of T cells in lymph nodes	[167]
Hydrogel	Antigen polypeptide WT1126-134 and immune adjuvants (GM-CSF)	Leu	Induce local immune-cell infiltration and activate dendritic cells	[166]
LNT cells	DOX	Leu	Promote antitumor immune responses	[171]
HSCs	aPD-1	Leu	Increase the number of active T cells, produce cytokines and chemokines	[172]
Lipid-PEG	mRNA, palmitic acid-modified TLR7/8 agonist R848 (C16-R848)	Lyn	Improve the expansion of OVA specific CD8 + T cells	[168]
PLGA NPs	BCMA72 − 80[YLMFLLRKI] peptide	MM	Increase peptide delivery to human dendritic cells, which enhance induction of BCMA-specific CTL	[169]

*APC* Antigen-presenting cell, *WT1* Wilms tumor protein 1, *GM-CSF* Granulocyte–macrophage colony-stimulating factor, *LNT cells* Liquid nitrogen-treated cells, *DOX* Doxorubicin, *TLR* Toll-like receptors, *PLGA* Poly(lactic-co-glycolic acid), *BCMA* B cell maturation antigen

#### Improve car-T therapy

CAR-T therapy has made progress in the treatment of refractory and relapsed B cell leukemia and lymphoma. Due to the lack of suppressive cells in the TME, such as in solid tumors, CAR-T cells and immune cells can easily access cancer cells and then kill them. However, the preparation of CAR-T cells is complicated and costly and may cause serious CRS side effects, which seriously restricts their application. To simplify the preparation process, Smith et al*.* reported a method for preparing CAR-T cells in vivo by using nanoparticles [170]. This nanoparticle comprises a PGA polymer, which is modified with CD3 antibody for T cell targeting on the surface, and the plasmid which expressing chimeric antigen receptor was loaded inside. These nanoparticles can achieve in vivo editing and can substantially prolong the survival of mice in animal experiments.

### Improve diagnostic accuracy

The clinical diagnosis of HMs currently mainly relies on blood, bone marrow, cytochemical staining and labeling of monoclonal antibodies to detect leukemia cell surface differentiation antigens and other biochemical detection methods. Currently, there is still lack of sensitive and specific molecular biology detection methods. Unclear detection of leukemia leads to undertreatment or overtreatment and side effects, seriously affecting the patient’s survival.

The emergence of nanotechnology has made the diagnosis of tumors more accurate. With the help of the paramagnetic and fluorescent labels of nanoparticles, the location and progress of cancer cells can be accurately diagnosed. For example, a micro-nanodiagnostic instrument can be implanted in the human body to run with the blood, and real-time transmission of internal information to the external recording device. Nanotechnology can make the diagnosis of leukemia more rapid and accurate [32], thereby contributing to treatment and prognosis [173]. The early diagnosis of tumors, especially malignant tumors, is very important to help improve clinical diagnosis results and choose effective treatment methods.

Electrochemical-based nanotechnology is widely used in diagnosis of leukemia [174–177]. Hu et al*.* reported a gold nanoparticle-modified glassy carbon electrode (GCE) used to distinguish different leukemia cancer cells [178]. By changing the ratio of reagents and the deposition time, gold nanoparticles of different sizes can be deposited on the GCE, and the increase in the concentration of cysteine may cause the Au particles to increase significantly. The changes in the electrochemical behavior of the probes were detected on the Au NPs-modified GCE, and the changes in the electrochemical signal for different cancer cells can identify different target cells. This study provide a new strategy for the rapid identification of leukemia cells [178].

Antibody-modified nanoparticles can also be used to track tumor cells and improve diagnostic accuracy [179]. Chaudhuri et al*.* reported that a fluorescently labeled antibody-conjugated nanotube can be used to quickly and label-free detect CD45 + microvesicles from leukemia cells in about 30 min [180]. The detection principle is based on the molecular recognition between antigens and antibodies.

Magnetic multifunctional probes have broad prospects in the fields of biomedical research and diagnosis. Song et al*.* have developed a monoclonal antibody (mAb)-conjugated magnetic nanobiological probes (FMBMNs) to detect and isolate a single type of tumor cells [181]. The probe can sensitively and efficiently detect and separate a variety of tumor markers or tumor cells from complex samples.

Temperature responsive nanotechnology can also be used to detect leukemia cells. Gold nanorods are widely used in nano-biodiagnosis research. The high level of lysozyme expression is a characteristic sign of leukemia. Moghadam et al*.* reported a gold nanorod that can induce aggregation at high temperature for rapid visual detection of lysozyme in serum. The gold nanorods are functionalized by connecting nucleic acid aptamers targeting lysozyme. Exposure of the nanoprobe to nanomolar level of lysozyme will cause specific structure aggregation. This research shows that the use of thermally induced gold nanorod aggregates has a broad prospect as a nanodiagnostic technology [182].

## Nanomedicine strategies against HMs

Principle of design nanomedicine is to specifically target and eliminate cancer cells while minimizing side effects. Based on tumor antigens which are highly expressed on the surface of cancer cells, modifying antibodies, peptides or aptamers that specifically recognize tumor antigens on the surface of nanoparticles is the most common strategy for nanomedicine design. According to recently reported HMs nanomedicines [183–192], we categorized the nanomedicines into four strategies: a. targeting the TME; b. targeting cell surface antigens; c. targeting intracellular signaling; and d. overcoming the drug resistance and enhance chemotherapy effect. In this chapter, we will elaborate on these strategies.

### Targeted disease organs

#### Bone marrow

**Fig. 5:**
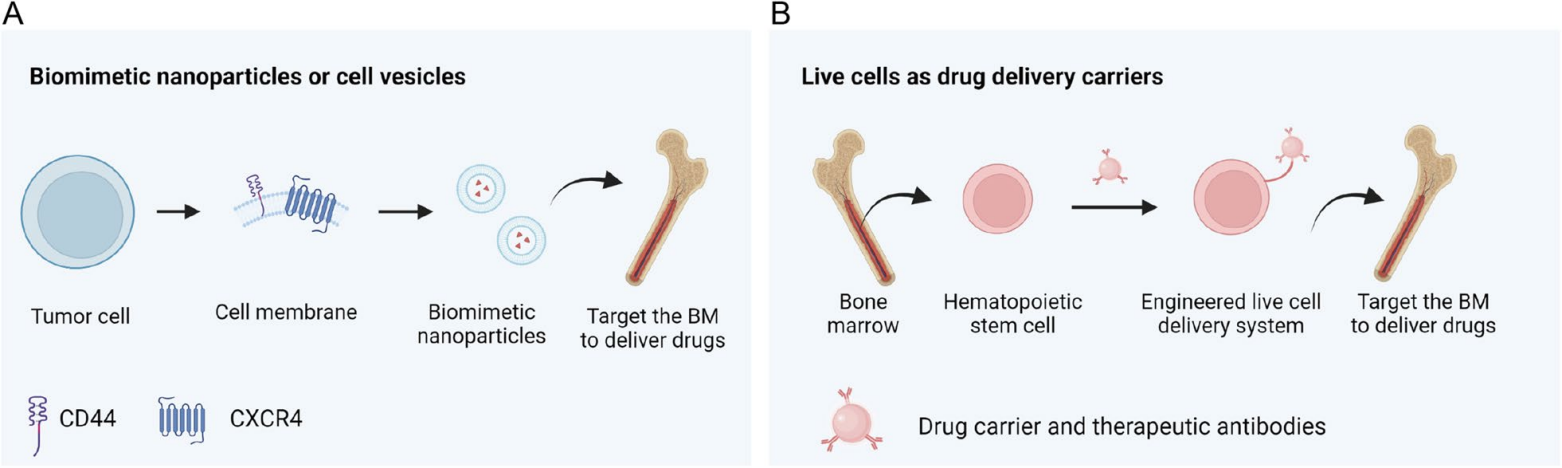
Strategies to target bone marrow microenvironment. **A** Cell membrane biomimetic nanoparticles are used to target bone marrow to deliver drugs. **B** Using the intrinsic bone marrow homing ability, living cells can be used as drug delivery carriers to target bone marrow for leukemia

The bone marrow is the main site for the pathogenesis and infiltration of leukemia and myeloma cells [193]. Residual cancer cells hidden in the bone marrow are the key factor for recurrence [194]. Thus, targeting bone marrow to deliver drugs is a promising strategy for the treatment of leukemia (Fig. 5). Hu et al*.* reported a strategy utilizing hematopoietic stem cells (HSCs) to deliver drugs to bone marrow [172]. In principle, taking advantage of the natural homing properties of HSCs to the bone marrow, platelets modified with a PD-1 antibody were attached on the surface of HSCs. The drug-loaded platelets targeted the bone marrow microenvironment along with HSCs and then released PD-1 drugs to enhance the effect of immunotherapy. In animal experiments, this drug considerably prolonged the survival of leukemic mice. Similarly, Ci et al. also reported a strategy that uses dead AML cells as carriers for the targeted delivery of drugs to the bone marrow [171]. This strategy uses liquid nitrogen-treated AML cells to generate a drug delivery system with bone marrow targeting properties, which improves the accumulation of chemotherapeutic drugs in the bone marrow and inhibits the progression of leukemia. Using the self-antigens of cancer cells combined with immune adjuvants, the immune cells can be stimulated to kill cancer cells. In animal experiments, this nanomedicine combined with immunotherapy substantially improved the survival time of leukemic mice. Biomimetic nanoparticles have considerable advantages in drug delivery. Dong et al. generated a biomimetic nanomedicine using the leukemia cell membrane for bone marrow drug delivery [195]. The drug has a core–shell structure and coated with NALM-6 leukemia cell membrane which were modified with TGFβRII antibody. The antibody is connected by a linker that responds to hypoxia and is inserted into the cell membrane. The core is an MSN structure carrying the chemotherapy drug DNR. The drug responds to the hypoxia signal in the bone marrow microenvironment of mice, then releases TGFβRII antibody and finally releases chemotherapeutic drugs to kill cancer cells. In a mouse model, the drug substantially prolonged the lifespan of leukemic mice. Similarly, myeloma cancer cell biomimetic nanoparticles loaded with drugs are also used in myeloma therapy [196].

#### Bone

Bone is the place where myeloma cells survive and plays an important role in the occurrence and relapse of MM. Targeting bone to delivery drugs is an effective strategy. Swami et al*.* reported an MM nanomedicine for targeting the bone microenvironment [197]. Similarly, Federico et al*.* reported a tumor microenvironment-targeted nanoparticle loaded with bortezomib and a ROCK inhibitor for the treatment of MM [198]. Stromal cells play an important role in the TME. Wang et al*.* generated a nanoparticle that dually targets myeloma cells and cancer-associated fibroblasts for treatment of MM [199]. Wu et al*.* constructed a bone-targeting nanoparticle for co-delivery of decitabine and arsenic in the treatment of MDS [200].

#### Lymph nodes

Lymph nodes (LNs) are the place where lymphocytes develop and mature, and also the place where lymphocytes transform into cancerous cells. Lymphoma has the characteristics of solid tumors, such as the presence of the immunosuppressive microenvironment and a high level of inflammation. Thus, targeted LNs delivery of drugs is an effective strategy for targeted elimination of lymphoma cancer cells. Schudel et al*.* reported a nanomedicine that can target the lymph nodes and can considerably inhibit the growth of lymphoma [201].

### Targeting cancer cells

**Fig. 6:**
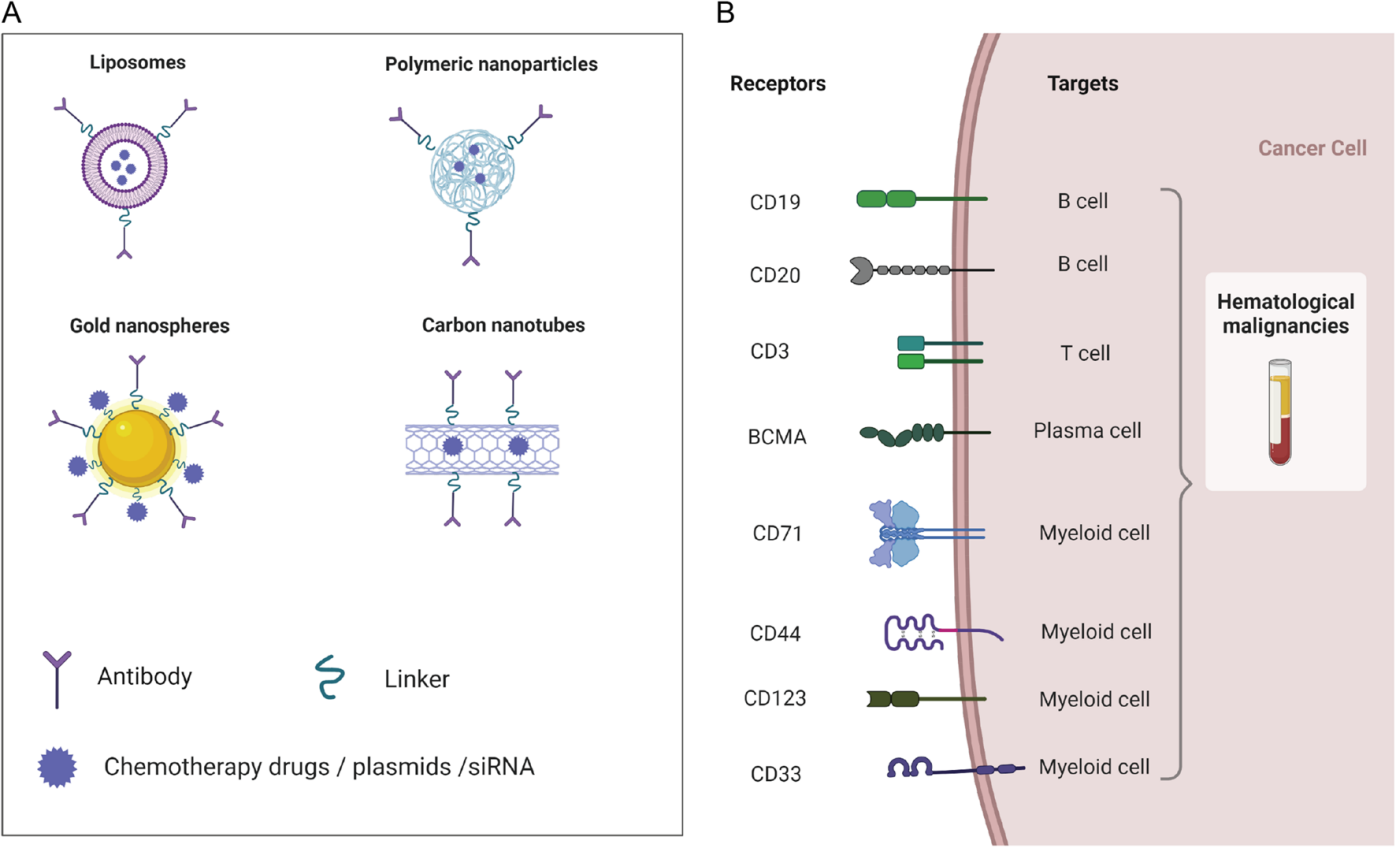
Targeting the surface antigens. **A** Drug delivery platforms against HMs. **B** Representative cell surface targets of HMs nanomedicine

Targeting the surface antigens of cancer cells is the most basic nanomedicine design strategy (Fig. 6). According to the biological functions of surface antigen, we divide leukemia surface antigens into three categories: (1) metabolism-related antigens, such as CD71 [202], FA receptor [203], PTK7 [204]; (2) immune antigens, such as CD19 [58, 205, 206], CD3 [207], CD33 [128, 208], B220 [209], CD117 [210], CD123 [211] and IL-1RAP [212], and (3) cell adhesion or migration-associated antigens, such as ITGB2 [93], CXCR4 [213] and CD44 [64]. The following is a detailed description of the above-mentioned antigen (Table 4).

**Table 4:** Representative nanomedicine targets and design of HMs

Targets	Carrier	Drug	Ind.	Outcome	References
CD19	Liposome	DOX	ALL	Significantly prolonged the survival of mice	[205]
	LNP	C61	ALL	Consistently caused apoptosis in B-precursor ALL cells	[58]
	Au NPs	NB	ALL	Proved superior cytotoxic effect against CCRF-SB cells	[206]
CD3	Gelatin NPs		ALL	Significantly increased cell absorption and internalization	[207]
B220	MSN	DNR	AML	Efficiently incorporated into and preferentially kill LSCs	[209]
CD117	CPSNPs	ICG	CML	In vivo efficacy of PDT was dramatically enhanced	[210]
CD123	PMBN	PTX/GA-A	AML	Increased anti proliferation of cells	[211]
	CdTe QDs	DNR	MDS	Effectively inhibited the tumor growth of MDS-bearing nude mice	[216]
IL-1RAP	Cas9 RNP	MSCM-NF	AML	Reduced LSC colony-forming capacity and leukemic burden	[212]
CD71	Fn	Arsenic	AML	Exerted strong antileukemia effects in diverse xenograft models	[202]
PTK7	MS2	Porphyrins	AML	Killed Jurkat cells selectively even when mixed with erythrocytes	[204]
CXCR4	Micelle	Peptide E5	AML	Significantly inhibited the engraftment of leukemic cells in spleen and BM	[213]
CD44	PLGA	PTL	AML	Improved the bioavailability and selective targeting of leukemic cells	[64]
FA	CS	6-MP	ALL	Significantly elevated tumor intracellular drug release	[203]
CD40	PLGA	MTV	ALL	Significantly reduced tumor volume with increased caspase-3 activity	[231]
CD20	PLGA	AZD-2014	Lyn	Significantly improved efficacy of AZD-2014 against NHL cells	[220]
CD22	NK-92MI	Sialyl Lewis X	Lyn	Exhibited significantly enhanced tumor cell binding and killing	[232]
CD38	PBCL	S3I-1757	MM	Significantly reduced the tumor size by fourfold compared to S3I-NP	[227]
PDGFR-β	PLA	PTX	MM	Simultaneous clearance of CAF cells and MM tumor cells	[199]

*PTX* Paclitaxel, *C61* Spleen tyrosine kinase (SYK) P-site inhibitor, *NB* Nile Blue, *DNR* Daunorubicin, Cas9 RNP Cas9/single guide RNA (sgRNA) ribonucleoprotein [lipidoid nanoparticle (LNP)], *MSCM-NF* Mesenchymal stem cell membrane-coated nanofibril, *PTL* Parthenolide, *6-MP* 6-mercaptopurine, *CS* Chitosan, *PEO-b-PBC* Poly(ethyleneoxide)-block-poly(α-benzylcarboxylate-ε-caprolactone)

The leukemia cells highly and stably express the transferrin receptor CD71 on the cell membranes due to the high demand for iron and abnormal iron metabolism. Based on this trait, Ma et al*.* designed a high-affinity arsenic nanomedicine based on ferritin for specifically targeting leukemia cells [202]. This nanomedicine uses ferritin particles as a carrier for delivering the arsenic drug and achieves the same antileukemia effect while reducing the dosage of the arsenic. This result was observed in a preclinical study, and the actual effect needs to be clinically verified. Similarly, Macone et al*.* delivered cytochrome C using ferritin as nanocarriers for the treatment of APL leukemia with high expression of CD71 on cell surface [214].

Cancer stem cells (CSCs) play a key role in the pathogenesis and relapse [215]. Conventional chemotherapy and immunotherapy cannot completely eliminate the leukemia stem cells (LSCs) hidden in the bone marrow. Thus, targeted elimination of LSCs is the key to preventing recurrence and ultimately curing leukemia. The main targets of nanomedicines against LSC include CD33, B220, CD117, CD123, and IL-1RAP. Alambin et al*.* generated a nanodrug targeting CD123 molecules on the surface of LSCs [211]. This nanomedicine comprises a PMBN polymer, and the ligand IL-3 for binding to the CD123 receptor on the surface of LSCs. In the mouse model, this nanodrug can considerably prolong the survival of leukemia model mice. In addition to being a target for leukemia cells, CD123 can also be used as a target for MDS nanomedicine. Guo et al*.* reported a nanomedicine modified with CD123 antibody for the treatment of MDS [216]. The nanomedicine uses daunorubicin loaded CdTe quantum dots as a carrier, and the surface is modified with CD123 mAb for targeting high-risk MDS cells. Animal experiments showed that the nanomedicine could significantly reduce tumor burden. Mandal et al*.* reported a nanomedicine targeting the B220 antigen on the surface of AML stem cells [209]. This nanomedicine uses mesoporous silica nanoparticles (MSNs) as a carrier, which is modified with B220 antibody on the surface and loaded with the chemotherapeutic daunorubicin inside. In vitro experiments show that this nanomedicine (anti-B220 MSN-DN) can be effectively absorbed by leukemia cells and preferentially kill B220-positive AML stem cells. In vivo experiments showed that after short-term pre-treatment with this nanomedicine, the pathogenicity of AML stem cells was substantially reduced.

Leukemia stem cells prefer to live in the bone marrow. Ho et al*.* generated a nanomedicine for targeting leukemia stem cells in the bone marrow and delivered gene editing tools to silence oncogenes [212]. The nanomedicine was injected into the bone marrow of mice, targeted leukemia stem cells through CXCL12α-mediated chemotaxis and released the Cas9 plasmid and IL1-RAP sgRNA intracellularly to silence the gene. The nanomedicine can considerably prolong the survival time of mice in the leukemia mouse model. *Brian *et al*.* developed a PDT therapy via targeting leukemia stem cells using CPSNP calcium phosphate nanoparticles loaded with ICG [210]. Among them, ICG is used as a photosensitizer for leukemia PDT and specifically targets the surface molecules CD117 or CD96 of leukemia stem cells through a bio-conjugation method. In the leukemia mouse model, the in vivo therapeutic effect of PDT was significantly improved by using ICG-CPSNPs targeting CD117. Studies have shown that CPSNPs targeted to leukemia stem cells and loaded with ICG are expected to treat relapsed and multidrug-resistant leukemia.

In lymphoma, construction nanomedicines for targeting lymphoma cells are also getting a lot of attention. Generating nanomedicine specifically targeting lymphoma cells is the basic strategy. The targets of lymphoma nanomedicines mainly include CD20, CD40, folate receptor (FR) and BCR. Qiu et al*.* generated a nanodrug targeting folate receptors (FR) on the surface of lymphoma cells [217]. Similarly, Zhao et al*.* generated a biomimetic silver nanoparticle for targeting FA receptors on lymphoma cells [218]. Regarding other targets, Nevala et al*.* reported an CD20 antibody-modified paclitaxel-loaded nanoparticle for the treatment of CD20 + B cell lymphoma [219]. Tang et al*.* reported a rituximab (anti-CD20)-modified AZD-2014 encapsulated nanoparticle for killing B lymphoma cells [220]. Torino et al*.* reported a BCR-targeted multimodal imaging-engineered nanoparticle for the therapeutic and diagnosis of B cell lymphoma [221].

In other blood cancer, nanomedicine studies have also increased in recent years [222–224]. Based on the highly expressed antigens on the surface of myeloma cells, the targets of MM nanomedicines mainly include CD38, folate receptor (FR) and VLA4. Omstead et al*.* first performed an in vivo evaluation of use CD38 and CD138 as targets for nanoparticle-based drug delivery in MM [225]. Puente et al*.* reported a CD38-targeting nanoparticle for enhancing bortezomib activity and the specificity of proteasome inhibition in MM [226]. Huang et al*.* showed that modification of anti-CD38 on nanoparticles carrying STAT3 inhibitors can improve the therapeutic effect of myeloma [227]. Similarly, Yu et al*.* generated a CD38-targeted daratumumab immunopolymer for chemotherapy of MM [228]. Regarding other targets, Fontana et al*.* reported a VLA4-targeted nanoparticle for the treatment of MM that functioned by hijacking cell adhesion-mediated drug resistance [229]. Nigro et al*.* generated bortezomib-loaded mesoporous silica nanoparticles (MSNs) targeting FA receptors to selectively alter metabolism and induce the death of myeloma cells [230].

### Targeting intracellular signaling pathways

Abnormal activation of intracellular signaling is an important signature of cancer cells. In CML cells, the BCR-ABL fusion gene is continuously highly expressed. This gene encodes a tyrosine kinase, which plays a key role in maintaining the survival of leukemia cells. In this context, Liu et al*.* reported a nanomedicine for targeted degradation of the BCR-ABL fusion gene in CML cells [233]. In an animal model, this nanomedicine could substantially prolong the survival of mice. Similarly, Vinhas et al*.* designed an Au nanoparticle-based AuNP@PEG@e14a2 for silencing BCR-ABL fusion gene overexpressed in CML [234].

Mutations of genes involved in epigenetic pathways play an important role in the pathogenesis of leukemia. The Bim1 gene has been reported to maintain the stability of histones in the nucleus. Kushwaha et al*.* reported a nanomedicine for targeting and degradation of Bmi1 mRNA in leukemia cells. The drug uses PEI and has as nanocarriers and encapsulates si-Bmi1 for Bmi1 gene. In animal experiments, this nanomedicine substantially prolonged the survival of diseased mice [235]. Similarly, Chandran et al*.* reported a study using HSA as nanocarriers to deliver HDAC inhibitors for AML treatment [236].

Besides specific signaling molecules, abnormal activation of pathways also plays an important role in cancer cell survival. Deng et al*.* designed a Au nanomedicine to target the NCL/miR-221/NF-kB/DNMT1 pathway in leukemia cells [237]. In mouse experiments, this nanomedicine could considerably prolong the lifespan of leukemic mice. Similarly, Dash et al*.* generated a nanomedicine that can trigger apoptosis mediated by the ROS/TNF-α pathway in leukemia cells [238].

**Fig. 7:**
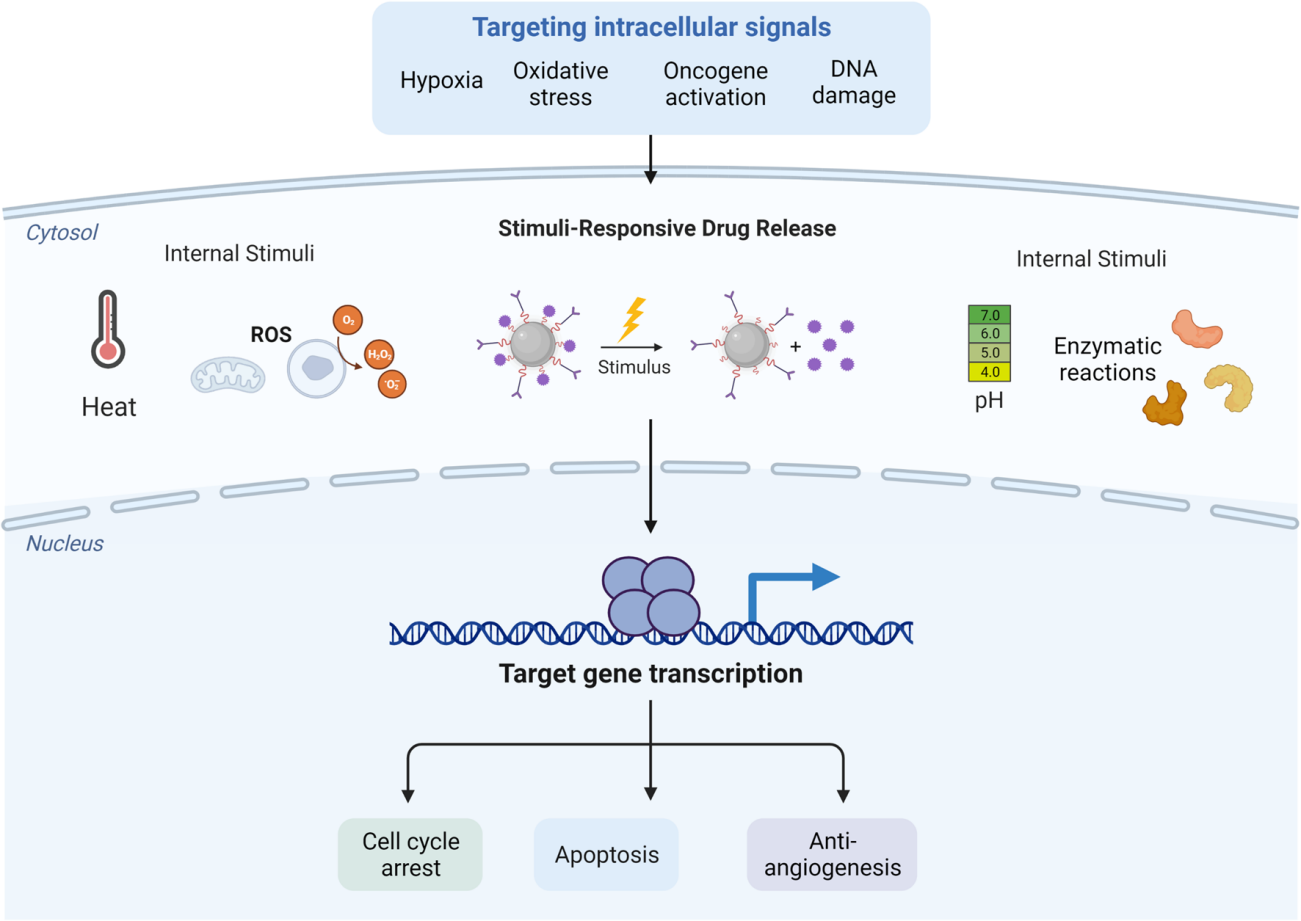
Targeting the intracellular signaling pathways

Besides cell surface antigens, intracellular signaling molecules and metabolic pathways, such as Ara-KB, hypoxia and autophagy pathways, can also be used as targets to design nanomedicines (Fig. 7). Li et al*.* generated a tissue factor-targeted “O_2_-Evolving” nanoparticle for photodynamic therapy of malignant lymphoma [239]. AZD2811 is an aurora kinase B inhibitor that disrupts cell mitosis. Its precursor, AZD1152, has shown promising results in clinical trials against acute myeloid leukemia, but with severe myelosuppression side effects. So the researchers developed Accurins, a nanodrug that contains different concentrations of AZD2811. Floc'h et al*.* proved that the nanoparticle formulation of AZD2811 had stronger anti-B cell tumor effect [240]. Martucci et al*.* have developed a nanodrug targeting BCL-2 for the treatment of B cell lymphoma [241]. Lin et al*.* generated an iron oxide nanoparticle that could remotely and magnetically control the autophagy process in mouse B lymphoma cells [242]. Adjuvants play an important role in cancer treatment. Lin et al*.* reported that preparing the adjuvant into the form of nanoparticles can enhance the killing effect of lymphoma cells [243]. Shadab et al*.* demonstrated that asparagine-laminated gold nanoparticles (Asn-AuNP conjugates) can enhance toxicity to leukemia cells by targeted intracellular heat transfer [244].

### Enhanced pharmacokinetics

#### Reverse drug resistance

Drug resistance in cancer cells is an important factor leading to poor chemotherapy outcome and cancer recurrence. Increasing the uptake of drugs in cancer cells is an effective strategy to improve the tumor killing effect. Nanoparticles loaded with chemotherapeutic drugs can considerably increase the uptake and accumulation of drugs in cells and enhance the therapeutic efficacy of free chemotherapy drugs [245, 246].

Ion channel proteins on cell surface play a key role in mediating drug resistance. The P-glycoprotein plays an important role in the drug resistance of cancer cells. Li et al*.* reported a strategy to use carbon nanotubes to overcome leukemia drug resistance [87]. The drug uses single-walled carbon nanotubes as a carrier, modified the P-gp antibody on the surface of the carbon nanotubes and encapsulated the chemotherapeutic drug DOX inside the nanotubes. In vitro results confirmed that this strategy can significantly increase the accumulation of Dox in K562 cells, enhance the toxicity to cancer cells and overcome cell resistance. Similarly, Zhang et al*.* designed a PLA polymer nanoparticle surface modified with P-glycoprotein antibody to overcome AML resistance and improve the therapeutic effect [247]. In addition, Song et al*.* reported that modification of the chemotherapeutic drug DNR on the surface of TiO_2_ nanoparticles by electrostatic adsorption can drastically increase the accumulation of DNR in K562 cells and reduce P-glycoprotein-mediated drug resistance [124].

The role of the ABC protein family in mediating drug resistance has been widely reported. Man et al*.* reported that modifying DNRs on the surface of nanodiamonds can bypass ABC transporter-mediated drug resistance and increase the accumulation and toxicity of DNRs in cancer cells [91].

Organic polymer materials and biological macromolecules are also used as nanocarriers to load chemotherapeutics to overcome drug resistance. Guo et al*.* loaded the chemotherapeutic drug DNR and the photosensitive molecule NIR797 into PEG-PLL-PLGA polymer nanoparticles [66]. In vitro experiments proved that the nanoparticles can substantially increase the toxicity to leukemia cells. Albumin aggregates are widely used as drug delivery vehicles in cancer research. Wu et al*.* designed an albumin-based nanodrug system to increase drug load and achieve controlled release of Doxorubicin [248]. This drug can enhance the accumulation of DOX drugs in cells and overcome drug resistance. Similarly, Kayani et al*.* reported that used bovine serum albumin nanoparticles as delivery carriers and encapsulated Dox to prepare DOX-DBSA-NPs can enhance the killing effect of drugs on leukemia cells and reverse drug resistance [249].

#### Improving the chemotherapy

Enhancing the drug toxicity to cancer cells is also an effective strategy for enhancing the therapeutic effect. We selected representative nanomedicine to demonstrate their better pharmacokinetics and therapeutic effects compare to non-nanomedicine (Table 5).

**Table 5:** Comparison between nanomedicine and non-nanomedicine against HMs

Drugs	Carrier	Outcomes compared with non-nanomedicine or free drugs	Ind.	References
Ara-C, DNR	Liposome	Exhibited potent and direct ex vivo cytotoxicity against AML blasts	Leu	[39]
VCR	Liposome	Improved the pharmacokinetics and pharmacodynamics of vincristine	Leu	[38]
C6-ceramide	Liposome	Selective inhibition of the glycolytic pathway in CLL cells	CLL	[51]
DOX	AlPcS	Enhanced the cellular uptake of AlPcS three times and PDT therapy	Leu	[250]
Platinum	MSN	Exhibited unprecedented enhanced cytotoxicity to cancerous cells	Leu	[100]
DAC,BTZ	PEG-PCL	Good stability, slow release profile, and superior anticancer effects	MM	[253]
As_4_S_4_	Realgar NPs	Significantly depleted the stem-like proportion and clonogenicity	MM	[254]

*DAC* 5-Aza-2ʹ-deoxycytidine, *BTZ* Bortezomib, *VCR* Vincristine

Qin et al*.* proved that conjugation of photosensitizers sulfonated aluminum phthalocyanine with chemotherapy drugs DOX can significantly improve the efficacy of photodynamic therapy for leukemia [250]. Kim proved that conjugation of the macromolecular prodrug of doxorubicin with the biodegradable cyclophosphazene containing tetrapeptide can improve the drug treatment effect [251]. Similarly, hematoporphyrin–platinum(II) conjugates also enhance the killing effect of platinum drug against leukemia cells [252]. For other types of hematopoietic malignancies, Che et al*.* demonstrated that nanoparticles encapsulating both bortezomib and DAC significantly enhanced the toxicity to MM cells [253].

## Perspectives

### Enhancing CAR-T therapy through nanotechnology

Enhancing the function of CAR-T cells through nanotechnology is an extremely promising field in cancer treatment [255]. Nanotechnology needs to be explored to overcome or reduce the exhaustion and side effects of CAR-T therapy [256]. Nanoparticles can be attached to the surface of CAR-T cells to enhance the killing function of CAR-T cells. Tang et al*.* reported a TCR-signaling responsive nanoparticle to improve the killing of CAR-T cells [257]. Similarly, the use of click chemistry to attach cytokines to the cell surface can also be used to enhance CAR-T therapy [258]. Similarly, click chemistry was used to attach nanoparticles to the surface of CAR-T cells to enhance CAR-T cell killing. By linking ICG-loaded nanoparticles onto the surface of CAR-T cells, combined with photodynamic therapy, the immune barrier of solid tumor was destroyed, and the infiltration and antitumor effect of CAR-T cells were enhanced [259]. Similarly, by modifying anti-CD3/CD28 immunomagnetic beads on the surface of CAR-T cells, combined with magnetic–acoustic methods, the activation and proliferation of CAR-T cells are enhanced [260].

In addition to cell surface modification nanotechnology, the combination of engineered nanoparticles also enhanced CAR-T therapy. Li et al*.* reported that using genetically programmable vesicles to improve the tumor microenvironment and enhance the killing function of CAR-T cells [261].

Moreover, the drawbacks and shortcomings of CAR-T therapy also need to be addressed. CAR-T therapy possesses serious side effects, which can produce serious CRS and neurotoxicity [262]. In addition, the excessive cost and high price of CAR-T products and the poor financial capacity of most patients can be another obstacles in the clinic. The resistance to CAR-T therapy is also one of the issues that continues to be addressed [263–265]. Regarding therapeutic targets, currently, CAR-T therapy only is effective in treating B cell-derived cancer, such as B-ALL, B cell lymphoma and myeloma. Due to lack of specific targets, CAR-T therapy against myeloid leukemia is still in preclinical studies [266]. In addition, the combination therapies with CAR-T therapy have shown great promise in preclinical study. For example, the combination of demethylating drugs can enhance the toxicity of CD123 CAR-T therapy on AML cells [267].

In addition to adoptive T cell-based therapy, other immune cells can also be used in immunotherapy. Engineering NK cells into CAR-NK for cancer treatment also holds great promise [268, 269]. Macrophages play an important role in the pathogenesis of HMs [270]. Conversely, engineering macrophages into CAR-Macrophage also showed good tumor killing ability [271]. In the future, nanotechnology-assisted ACT therapy holds promise in clinical application. In summary, utilizing nanotechnology to enhance CAR-T therapy still has enormous untapped potential for researchers.

### Establishment of more effective and lasting immunological memory through nanotechnology

Relapse is the most serious problem affecting the prognosis and survival of HMs patients. Most patients will face recurrence after first remission. Studies on relapsed AML patients after allogeneic transplantation have shown that reducing MHC-II levels in cancer cells is an important cause leading to immune escape and relapse [272].

To achieve long-term tumor control, the immune system must be trained to form lasting memories [273]. Therefore, developing a robust and effective vaccine has great prospects and commercial value. Traditional vaccines usually lack of sufficient antigenic activity, and thus, the body cannot produce lasting immune memory [165]. In future research, new leukemia antigens will be identified through more extensive and comprehensive bioinformatics analysis and the more efficient antigen presentation will be realized by the use of nanocarriers. We can design nanomedicine according to the new therapeutic targets and antigens against HMs by enhancing the body’s immune response, ultimately achieving long-term control of HMs.

### Improving the effectiveness of HSCT through nanotechnology

Regarding HSCT, using nanomaterials to promote the expansion of HSCs is of great help to improve the efficiency of HSCT. It is critical to understand and determine the key chemical and physical conditions that promote the expansion of HSCs [145] and most importantly prevent the malignant transformation of HSCs into cancerous cells during ex vivo culture [150]. Establishing a suitable in vitro expansion system is the key to achieving this goal [274, 275]. Encouragingly, a large number of studies focusing on the ex vivo expansion of HSCs are being reported, showing great prospects in this field.

### Potential toxicity of nanomaterials to blood cells

Although nanomaterials show excellent properties and great prospects in drug delivery, the toxicity of various nanomaterials still need to be carefully studied [276, 277]. The following summarizes the toxicity of the metal and carbon nanomaterials mentioned above.

In addition to being drug delivery carriers, metal nanoparticles themselves can also cause toxicity to cells. *Tsai *et al*.* showed that after treatment with gold nanoparticles (Au NPs), human chronic myeloid leukemia cells showed growth inhibition and apoptotic necrosis phenotype [278]. Mechanistically, the analysis of proteomic data reveals that the unfolded protein-related endoplasmic reticulum (ER) stress response is the main event and Au NPs are an effective endoplasmic reticulum stress inducer. The toxicity of other common metal nanoparticles is shown in Table 6.

**Table 6:** Representative studies on the toxicity of nanoparticles to blood cells

Nanoparticles	Object cell	Toxicity	Refs
Au NPs	K562 cells	Induced cell death by inducing ER stress	[278]
Au NPs	HL-60 cells	Induced caspases-dependent apoptosis	[282]
Ag NPs	HL-60 cells	Caused ROS mediated cytotoxicity	[283]
Ag NPs	Ba/F3 cell	Induced autophagy through the mTOR pathway mediated by ROS	[284]
PbO_2_ NPs	K562	Caused oxidative stress, cytotoxicity and cell death	[285]
CNT	HL-60	Induced cell cycle arrest and apoptosis through AKT and MAPK pathways	[279]
	THP-1	The longer nanotubes induced a stronger inflammatory response	[280]
GFNs	THP-1a	The surface oxidation states may cause GFNs to behave differently and cause different immunotoxic effects	[281]

*CNT* carbon nanotube, *GFNs* graphene family nanomaterials

Carbon-based nanomaterials are also toxic to cells. Dinicola et al*.* showed that multi-walled carbon nanotube buckypaper can induce cell cycle arrest by regulating AKT and MAPK signaling pathways and raised the issue of biocompatibility and potential toxicity [279]. Sato et al*.* investigated the cytotoxicity of carbon nanotubes of different lengths to monocytes and found that longer nanotubes were more toxic because they were harder for macrophages to envelop [280]. Similarly, Yan and colleagues have continuously evaluated the effects of GONPs and rGONPs on THP-1 and THP-1a, proving that the surface oxidation state may lead to different expressions of GFN and different immune toxicities [281].

## Conclusions

In the end, nanomedicine is gradually moving out of the laboratory and entering clinical trials, and there are also many problems to be faced [286]. The biological safety of nanomedicine has gradually attracted people’s attention. The main problems of nanomedicine entering the clinic are: a. preparation problems. Most of the nanomedicines that have been reported are in the laboratory research stage, and the large-scale preparation of nanomedicine needs to be carried out in factories, and the current factories generally lack corresponding production lines. b. Biological safety issues. As a new type of medical materials, nanomaterials still have other unknown side effects when taken as drugs. More laboratory and clinical trials are needed to ensure the safety of nanomedicine. c. Cost issues. As an emerging technology, nanotechnology generally has a relatively high production cost. If it becomes a clinical first-line drug, the production cost should be reduced so that the medical systems and patients of various countries can afford it.

## Data Availability

Not applicable.

## Abbreviations

6-MP: 6-Mercaptopurine
APC: Antigen-presenting cell
BCMA: B cell maturation antigen
BTZ: Bortezomib
C61: Spleen tyrosine kinase (SYK) P-site inhibitor
Cas9 RNP: Cas9/single guide RNA (sgRNA) ribonucleoprotein [lipidoid nanoparticle (LNP)]
CNT: Carbon nanotube
CS: Chitosan
DAC: 5-Aza-2ʹ-deoxycytidine
DNR: Daunorubicin
DOX: Doxorubicin
GFNs: Graphene family nanomaterials
GM-CSF: Granulocyte-macrophage colony-stimulating factor
HCL: Hydrochloride
HSCs: Hematopoietic stem cells
LNT cells: Liquid nitrogen-treated cells
MSCM-NF: Mesenchymal stem cell membrane-coated nanofibril
NB: Nile Blue
PEO-b-PBC: Poly(ethyleneoxide)-block-poly(α-benzylcarboxylate-ε-caprolactone)
PGA: Poly (glutamic acid)
PLA NPs: Poly (lactic acid) nanoparticles
PLGA: Poly(lactic-co-glycolic acid)
PTL: Parthenolide
PTX: Paclitaxel
TLR: Toll-like receptors
TME: Tumor microenvironment
VCR: Vincristine
WT1: Wilms tumor protein 1
